# Case Report: Dacomitinib May Not Benefit Patients Who Develop Rare Compound Mutations After Later-Line Osimertinib Treatment

**DOI:** 10.3389/fonc.2021.649843

**Published:** 2021-04-15

**Authors:** Hong-Shuai Li, Guang-Jian Yang, Yan Wang

**Affiliations:** Department of Medical Oncology, National Cancer Center/National Clinical Research Center for Cancer/Cancer Hospital, Chinese Academy of Medical Sciences and Peking Union Medical College, Beijing, China

**Keywords:** osimertinib, resistance, dacomitinib, L792, L718

## Abstract

The acquired *EGFR* C797X mutation has been identified as the most notable resistance to osimertinib, and novel secondary mutations of *EGFR* L718 and L792 residues have also been demonstrated to confer osimertinib resistance, making the choice of medication after osimertinib treatment a quandary. Dacomitinib has been reported to have potential impact on patients acquiring rare compound mutations after osimertinib resistance; however, little evidence is available to date. In five lung adenocarcinoma patients resistant to later-line osimertinib, recurrent mutations at *EGFR* L792 and/or L718 were identified using targeted next-generation sequencing of tissue or cell-free DNA from plasma or pleural effusion. Dacomitinib was initiated after osimertinib resistance; however, all patients progressed within 2 months. Molecular structural simulation revealed that L792H + T790M and L718Q mutations could interfere with the binding of dacomitinib to EGFR and potentially cause primary drug resistance. Our case series study, to our knowledge, is the first to report the clinical efficacy of dacomitinib in patients harboring rare complex mutations after later-line osimertinib resistance.

## Introduction

Non-small cell lung cancers (NSCLCs) that harboring tyrosine kinase inhibitor (TKI)-sensitive epidermal growth factor receptor (*EGFR*) mutations show remarkable initial response to EGFR-TKIs; however, majority of patients develop resistance and undergo progressive disease. The third-generation (3G) EGFR-TKI osimertinib, initially approved as the second-line treatment for patients with T790-mutant NSCLC, is standard of care for patients with *EGFR*-mutated NSCLC; however, it has the same dilemma as that of prior generations of TKIs, limiting its progression-free survival (PFS) to 10.1 months in the later-line treatment (1).

The acquisition of *EGFR* C797X mutation has been identified as the most notable resistance mechanism that abolishes the covalent binding of osimertinib to EGFR. Moreover, novel mutations of *EGFR* L718 and L792 residues have also been demonstrated to confer osimertinib resistance both *in vitro* and *in vivo* (2–4).* A* cohort study of 93 NSCLC patients resistant to second-line osimertinib using post-progression samples revealed that *EGFR* G796/C797, L792, and L718/G719 mutations were identified in 24.7%, 10.8%, and 9.7% of the cases, respectively (2). Most of the L792X mutations (10 of 11) coexist with other secondary *EGFR* mutations and are always cis with T790M mutations. In addition, the L792 substitutions were very diverse and multiple substitution types were observed in L792 in the same patient, with L792H ranking highest (2). L718Q is nearly as resistant to osimertinib as C797S, especially when in *cis* with the L858R mutation, indicating another independent drug resistance mechanism (2, 5). Compound mutations defined as double or triple mutations in the EGFR kinase domain are related to poor clinical outcomes (6, 7). However, to date, no optimal regimen has been accepted as a standard for this subset of patients.

Three approved EGFR-TKIs (erlotinib, afatinib, and osimertinib), or a combination of EAI045 and cetuximab, have shown no drug sensitivity to Ba/F3 cells, stably expressing *EGFR* L858R and T790M mutations in *cis* with the L792H mutation (4). Nevertheless, an *in vitro* experiment conducted by Nishino et al. (5) revealed that the 2 G EGFR-TKI (dacomitinib/afatinib) was effective for L718Q/V- or L792F/H-mutated Ba/F3 cells.

Dacomitinib is a potent, irreversible, highly selective, second-generation EGFR-TKI that inhibits signaling from both heterodimers and homodimers of all members of the human epidermal growth factor receptor family. The ARCHER 1050 trial laid the foundation for its use as a standard first-line option in patients with advanced *EGFR*-mutated NSCLC. In addition, dacomitinib appears to have potential application for patients acquiring compound mutations after osimertinib resistance; however, little evidence is available to date.

In this study, we present five cases of *EGFR*-mutated NSCLC with compound mutations after later-line osimertinib that failed to respond to dacomitinib.

## Cases Presentation

**Figure 1:**
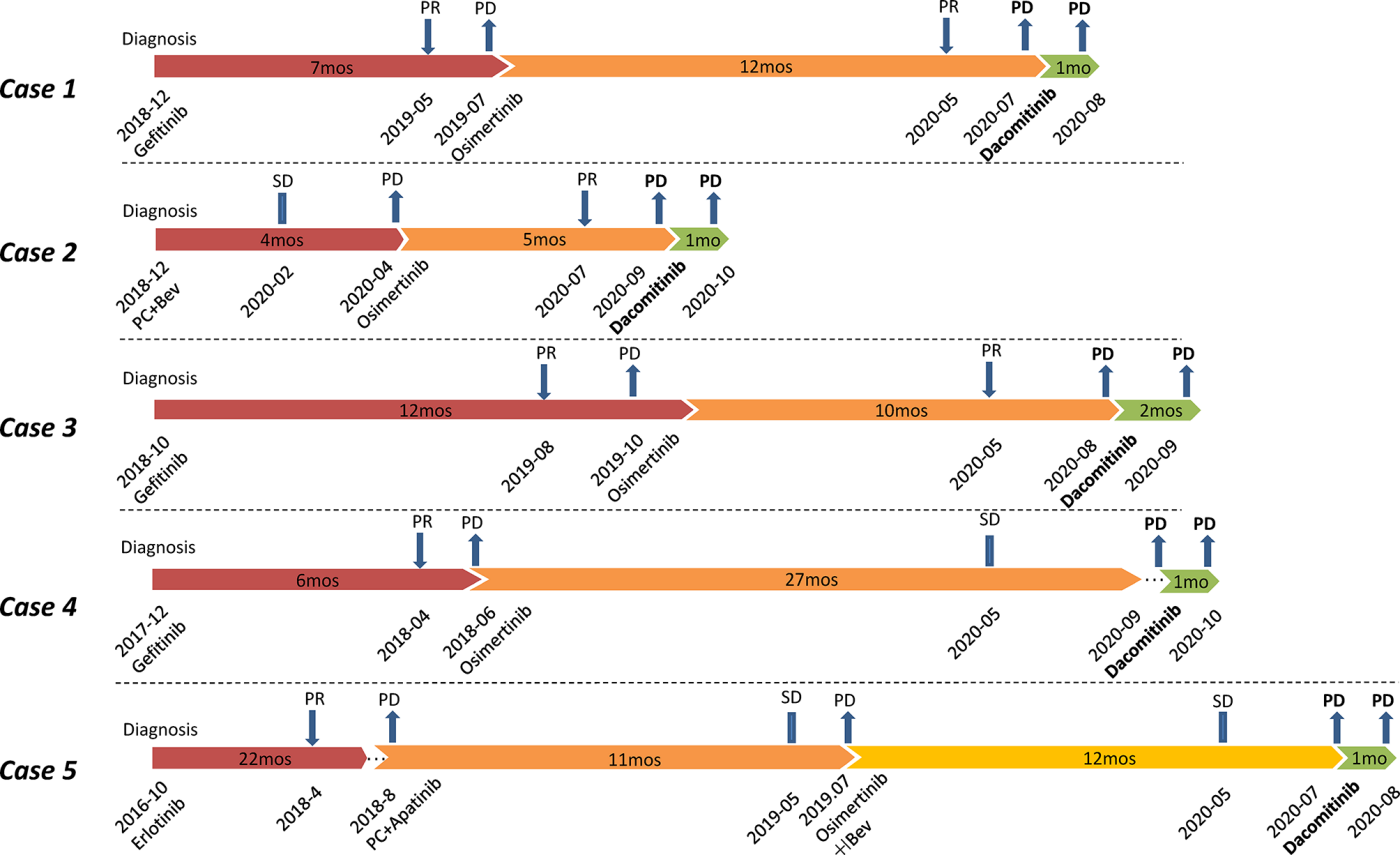
Flow chart of treatments for Case 1-5. All patients were treated with dacomitinib after osimertinib resistance. PR, partial response; SD, stable disease; PD, progressive disease; PC, pemetrexed plus carboplatin; Bev, bevacizumab; mos, months.

All the five patients in this study were diagnosed with stage IV lung adenocarcinoma, and prior 1G TKI gefitinib/erlotinib treatment and/or chemotherapy were administered before osimertinib (**Figure 1**). Plasma cell-free DNA (cfDNA) samples from all cases before osimertinib treatment were tested using targeted next-generation sequencing (NGS) (**Table 1**) (**Supplementary Table 1**). All cases achieved different levels of tumor reduction to osimertinib before progression, and the effects were maintained for 12, 5, 10, 27, and 12 months, respectively. At the time of systemic progression, plasma samples from Case 1–4, pleural effusions from Case 4, and liver samples from Case 5 were collected for cfDNA or tumor DNA extraction and subsequently examined using next-generation sequencing (NGS) (mean coverage depth >2500×) (**Table 1**), which was performed by qualified third-party genetic testing companies that had been accredited by the College of American Pathologists (CAP).

**Table 1:** Patients characteristics and *EGFR* mutation profile.

Case	Gender	Age	Smoking history	ECOG status	EGFR initial mutation (persistence at the progression to osimertinib) (abundance)	p.T790M status at the progression to osimertinib (abundance)	Progressive organ at the progression to osimertinib	Resistance mechanism to osimertinib (abundance)	NGS sample type
***Sequential 1G TKI → osimertinib group***									
1	F	38	N	1	p.L858R (Y) (12.5%)	Remained (3.5%)	Lung	p.L792F/H (8.7%)	Blood
3	M	58	Y	1	p.L858R (Y) (2.7%)	Lost	Lung	p.L718V (7.5%), L792V (3.5%)	Blood
4	F	72	N	1	p.L858R (Y) (0.6%)/(43.2%)	Lost	Peritoneum	p.L718Q (0.3%)/(0%)	Blood/pleural effusion
5	M	81	Y	2	p.L858R (Y) (0.5%)	Remained (0.3%)	Lung	p.L792H (1.2%)	Liver tissue
***Sequential chemotherapy → osimertinib group***									
2	M	45	Y	1	p.L858R (Y) (10.9%)	NA	Liver	p.L718Q (2.1%)	Blood

F, female; M, male; Y, yes; N, no; ECOG, Eastern Cooperative Oncology Group; NA, not applicable; NGS, next-generation sequencing.

### Sequential 1G TKI → Osimertinib Group

A total of four (Case 1, 3-5) of the five patients were treated with 1G TKI (gefitinib/erlotinib) followed by osimertinib. Upon resistance to osimertinib, NGS testing revealed not only the original *EGFR* L858R mutation at diagnosis with or without secondary T790M but also the novel mutations on L718 (L718Q/V) and/or L792 (L792F/H/V) (**Table 1**). Interestingly, although not specifically selected, the initial mutation in all these patients was L858R and they were all free of the most common C797S mutation after osimertinib treatment. For Case1 and Case 5, T790M was identified besides L858R by plasma and liver tissue, respectively. For Case 3–4, blood NGS revealed no T790M but L718X mutation. For Case 4, we obtained paired plasma and pleural fluid specimens from the patient. NGS results for both samples suggested presence of *EGFR* L858R and absence of *EGFR* T790M, but L718Q was only detected in the plasma sample.

All four patients were treated with dacomitinib after osimertinib resistance at an initial dose of 45 mg po qd. Two patients (Cases 1 and 5) had their treatment tapered to 30 mg po qd owing to intolerable diarrhea during medication. Imaging evaluation (computerized tomography and/or magnetic resonance imaging), 1 month after medication, suggested progressive disease (PD) owing to increased pulmonary, peritoneal, and pulmonary metastases in Cases 1, 4, and 5, respectively. For Case 3, an imaging re-check in the second month revealed PD owing to increasingly enlarged lung lesions.

### Sequential Chemotherapy → Osimertinib Group

A patient treated with osimertinib after chemotherapy progression experienced PD after achieving 5 months of PFS. Blood NGS testing suggested *EGFR* L858R and L718Q mutations (**Table 1**). He was then treated with dacomitinib after osimertinib resistance at an initial dose of 45 mg po qd. Unfortunately, imaging evaluation suggested PD owing to increased liver only 1 month later.

### Molecular Dynamics Simulation

**Figure 2:**
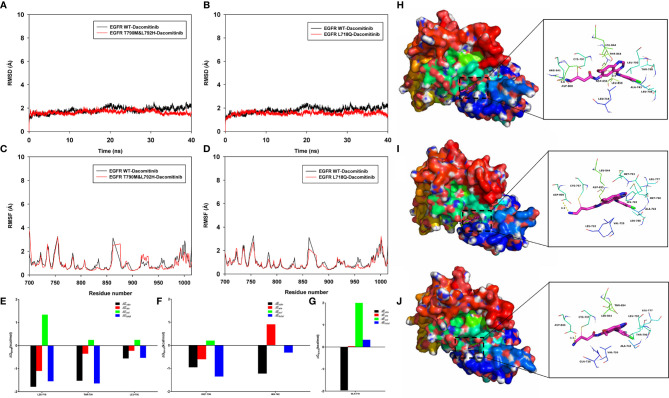
Molecular dynamics simulation. The root-mean-square deviation (RMSD) values of the protein backbone based on the starting structure along the simulation time were calculated and plotted in **(A, B)**, and the protein structures of the three systems (EGFR WT-Dacomitinib, EGFR T790M&L792H-Dacomitinib, EGFR L718Q-Dacomitinib with Dacomitinib) were stabilized during the 40-ns simulation. The root mean square fluctuations (RMSF) of residues are shown in **(C, D)**. The summations of the per residue interaction free energies were separated into *Van der Waals* (Δ*E_vdw_*) (black), solvation (Δ*E_sol_*) (green), electrostatic (Δ*E_ele_*) (red) and total contribution (Δ*E_total_*) (blue). In the EGFR WT-Dacomitinib complex, the residues L718 and T790 with the Δ*E_vdw_* of < -1.5 kcal/mol **(E)**, have an appreciable *Van der Waals* interactions with the Dacomitinib because of the close proximity between the residues and the Dacomitinib **(H)**. In the EGFR T790M&L792H-Dacomitinib complex, the residues M790 and H792 decomposed energy interaction originated from *Van der Waals* interactions, and the total contribution were both higher than that of EGFR WT **(F, I)**. In the EGFR L718Q-Dacomitinib complex, the residue Q718 decomposed energy interaction originated from *Van der Waals* interactions, and the total contribution was higher than that of EGFR WT **(G, J)**.

To explore the potential binding mode between the Dacomitinib and the EGFR, molecular dynamics simulations were performed using the Amber 14 software package. Detailed operation steps and methods can be found in the **Supplementary Material**. The binding mechanism of wild-type EGFR (EGFR WT), EGFR T790M&L792H and EGFR L718Q with Dacomitinib were determined by 40-ns molecular dynamics simulations. To explore the dynamic stability of the complex and to ensure the rationality of the sampling strategy, the root-mean-square deviation (RMSD) values of the protein backbone based on the starting structure along the simulation time were calculated and plotted in **Figures 2A**, **B**. As shown in **Figures 2A**, **B**, the protein structures of the three systems were stabilized during the 40-ns simulation.

The root mean square fluctuations (RMSF) of the residues of the whole protein in the EGFR WT-Dacomitinib, EGFR T790M&L792H-Dacomitinib and EGFR L718Q-Dacomitinib complex were calculated to reveal the flexibility of the residues. The RMSF of these residues are shown in **Figures 2C**, **D**, clearly depicting different flexibilities in the binding site of EGFR and the mutants in the presence of the Dacomitinib. The majority of the residues in the EGFR mutants binding site that bind with Dacomitinib showed a small degree of flexibility with a RMSF of less than 2 Å when compared with the EGFR WT, indicating that these residues seem to be more rigid as a result of binding to Dacomitinib.

To gain more information about the residues surrounding the binding site and their contribution to the system, the electrostatic, *Van der Waals*, solvation, and total contribution of the residues to the binding free energy were calculated with the MMGBSA method. The summations of the per residue interaction free energies were separated into *Van der Waals* (Δ*E_vdw_*), solvation (Δ*E_sol_*), electrostatic (Δ*E_ele_*) and total contribution (Δ*E_total_*). In the EGFR WT-Dacomitinib complex, the residues L718 and T790 with the Δ*E_vdw_* of < -1.5 kcal/mol (**Figure 2E**), have an appreciable *Van der Waals* interactions with the Dacomitinib because of the close proximity between the residues and the Dacomitinib (**Figure 2H**). Except for the residues L718 and T790, the residue decomposed energy interaction originated from *Van der Waals* interactions, apparently through hydrophobic interaction (*i.e.* L792). In the EGFR T790M&L792H-Dacomitinib complex, the residues M790 and H792 decomposed energy interaction originated from *Van der Waals* interactions, and the total contribution Δ*E_total_* were both higher than that of EGFR WT (M790 Δ*E_total_*=-1.35 kcal/mol *vs* T790 Δ*E_total_*=-1.64 kcal/mol; H792 Δ*E_total_*=-0.31 kcal/mol *vs* L792 Δ*E_total_*=-0.53 kcal/mol) (**Figures 2F, I**). In the EGFR L718Q-Dacomitinib complex, the residue Q718 decomposed energy interaction originated from *Van der Waals* interactions, and the total contribution Δ*E_total_* was higher than that of EGFR WT (Gln-718 Δ*E_total_*=0.33 kcal/mol *vs* Leu-718 Δ*E_total_*=-1.55 kcal/mol) (**Figures 2G, J**). In addition, the total binding free energy for the EGFR WT-Dacomitinib, EGFR T790M&L792H-Dacomitinib and EGFR L718Q-Dacomitinib complex was calculated according to the MMGBSA approach, and the estimated Δ*G_bind_* of -41.15 kcal/mol, -40.80 kcal/mol and -40.04 kcal/mol were found for Dacomitinib, respectively, suggesting that the mutations can affect the binding between EGFR and Dacomitinib.

In summary, the above molecular simulations give us rational explanation of the interactions between Dacomitinib and EGFR, which provided valuable information for Dacomitinib resistance and further development of the EGFR inhibitors.

## Discussion

To date, the application of dacomitinib on rare compound mutations in osimertinib-resistant settings remains elusive because little clinical evidence is available. Our case series study, to our knowledge, is the first to report the clinical efficacy of dacomitinib in this subset of patients and tried to explain the efficacy in molecular level.

So far, mechanisms of resistance to osimertinib have been relatively well studied (8–12), which can be broadly grouped into *EGFR*-dependent (on-target) (like *EGFR* C797X mutation, etc.) or *EGFR*-independent (off-target) mechanisms (including amplification of *MET*, *HER2*, *PIK3CA* amplification, and other mutations in *BRAF*, *KRAS* and *PIK3CA*, and oncogenic fusion mutations in *FGFR3*, *RET* and *NTRK*), and more *EGFR*-dependent mutations occur in later-line osimertinib setting than in first-line. In details, on-target mechanisms of resistance to first-line osimertinib consists of C797X (7%), development of complex mutations such as L718Q + C797S (1%), L718Q + ex20ins (1%), and S768I (1%) without clearly co-existence of T790M (11). In contrast, on-target mechanisms of resistance to later-line osimertinib consists of C797X (15%), L792H/F+C797S (1%), L792H (1%), G796S (1%), L718Q (1%), and ex20ins (1%) (8).

In a cohort study conducted by Yang et al. (2), L792X and L718X mutations were identified in 10.8% (10/93) and 9.7% (9/93) of osimertinib-resistant NSCLC cases, but were higher than those in 28.9% (11/38) and 18.4% (7/38) of T790M-ratained NSCLC cases, respectively. In addition, most L792-mutated cases have a concomitant *cis* T790M mutation, which is consistent with previous studies (4, 5, 13). In our study, two of the three cases harboring L792X mutations retained the T790M mutation and were both in *cis* with T790M; however, the other three cases harboring L718X mutations were absent from T790M. Interestingly, the initial mutation in all patients was L858R, and they were all free of the most common C797S mutation/*MET* amplification after osimertinib treatment, suggesting that the L718X mutations have mechanisms of drug resistance independent of C797X.

The application of dacomitinib to rare mutations (e.g., exon 20 insertion, exon 20 S768I, exon 18 G719C) has gradually attracted attention (14). Kobayashi et al. (15) demonstrated, *via* an *in vitro* study, that dacomitinib showed a promising therapeutic effect on L792F-mutated afatinib-resistant Ba/F3 cells. Another *in vitro* experiment conducted by Nishino et al. (5) revealed that dacomitinib was effective against L792F/H- or L718Q/V-mutated Ba/F3 cells. However, it did not demonstrate the expected clinical efficacy in our current study. According to our molecular simulation findings, in the EGFR T790M&L792H-Dacomitinib complex, the residues M790 and H792 decomposed energy interaction originated from *Van der Waals* interactions, and the total contribution Δ*E_total_* were both higher than that of EGFR WT, suggesting the presence of the T790M and L792H co-mutation compromises the efficacy of dacomitinib. The L718 side-chain has contact with the phenyl ring of the drug in the crystal structure of the dacomitinib-bound wild-type EGFR tyrosine kinase domain (PDB ID 4I24). Therefore, substituting L718 with V or Q is highly likely to alter the mode or orientation of dacomitinib binding. In fact, our molecular simulation analysis revealed, in the EGFR L718Q-Dacomitinib complex, that the residue Q718 decomposed energy interaction originated from *Van der Waals* interactions, and the total contribution Δ*E_total_* was higher than that of EGFR WT. In addition, the total binding free energy calculated (Δ*G_bind_*) for EGFR T790M&L792H-Dacomitinib and EGFR L718Q-Dacomitinib complex were both higher than EGFR WT-Dacomitinib, suggesting a much higher drug concentration to suppress tumor growth, which explains why the cell-based assay produced good results unlike the clinical application in our study. Another issue that should not be overlooked is that the cell lines constructed in the cell assay were not obtained after multiplex treatments, but were rather constructed directly *via* mutagenesis, mimicking the first-line osimertinib treatment. Patients typically have more concomitant genetic mutations and greater tumor heterogeneity after multiple treatments, which is an important reason for the discrepancy between cell assays and clinical results.

Interestingly, afatinib, which is also a 2G TKI, showed good tumor control not only in *in vitro* cellular assays (5) on L718X/L792X mutations but also in clinical application of L718X mutation according to several case reports (**Supplementary Table 2**) (16–20). Molecular structural models showed that, unlike dacomitinib, no clashes or spatial conflicts between afatinib in its L858R-bound orientation and the L718Q/V side-chain were observed (17, 21). More clinical data are needed to confirm whether afatinib is effective for the L718X mutation.

Drug-resistant mutations are numerous and endless, and the successive use of targeted drugs is not the ultimate solution. For rare compound mutations such as C797S, L792H, and L718Q, prevention may be more important than treatment; for instance, a combination of different generations of TKIs, such as osimertinib combined with gefitinib, can reduce the generation of drug-resistant mutations including C797S (22). In addition, given the different mechanisms of action, chemotherapy may be superior to targeted drugs for these rare mutations (4). Another promising treatment option is re-challenge therapy with targeted drugs. The rationale lies in the ability to kill all mutational subclones with chemotherapy after initial osimertinib resistance, making re-challenge with osimertinib a possibility (23).

Our study had certain limitations. First, the number of cases included in the study was small, and the role of dacomitinib in this population needs to be further investigated. Second, NGS sequencing depth is limited, and there may be some co-mutations that were not found, which may have an impact on the efficacy of dacomitinib. In addition, possibility of small cell lung cancer transformation cannot be ruled out as well due to inaccessibility of re-biopsy, though no obvious neuron-specific enolase (NSE) elevation was observed in our cases. Third, osimertinib has been approved as first-line drug for *EGFR*-mutated NSCLC. All patients in this study were received osimertinib as later-line therapy. Therefore, it is thought that dacomitinib was not effective after treatment of 1G-TKI and osimertinib. We believe that it could be interesting to test dacomitinib in patients progressing to first-line osimertinib after developing L792X and/or L718X mutations as it was tested *in vitro*, where Nishino et al. introduced these mutations into Ba/F3 cells in cis with activating EGFR mutations but not with T790M (5). Finally, the inconsistencies between cell assays and clinical results are not thoroughly interpreted; thus, further research is required to elucidate them.

In summary, our case series suggests that dacomitinib may be ineffective in patients who develop L792X and L718X mutations after osimertinib resistance. More clinical data are needed to confirm whether 2G TKIs (dacomitinib and afatinib) have potential applications in this subset of patients.

## Data Availability Statement

The original contributions presented in the study are included in the article/**Supplementary Material**. Further inquiries can be directed to the corresponding author.

## Ethics Statement

The studies involving human participants were reviewed and approved by National Cancer Center/National Clinical Research Center for Cancer/Cancer Hospital, Chinese Academy of Medical Sciences, Peking Union Medical College. The patients/participants provided their written informed consent to participate in this study. Written informed consent was obtained from the individual(s) for the publication of any potentially identifiable images or data included in this article.

## Author Contributions

H-SL: manuscript writing, provision of study materials or patients, and data analysis and interpretation. G-JY: manuscript writing and collection and assembly of data. YW: manuscript writing and conception and design. All authors contributed to the article and approved the submitted version.

## Funding

This work was supported by the Chinese Academy of Medical Sciences (CAMS) Innovation Fund for Medical Sciences (CIFMS) (2019-I2M-2-003) and Chinese Society of Clinical Oncology (CSCO)-Hengrui Research Funding (Y-HR2018-239).

## Conflict of Interest

The authors declare that the research was conducted in the absence of any commercial or financial relationships that could be construed as a potential conflict of interest.
