# Supplementary material for: Case Report: Dacomitinib May Not Benefit Patients Who Develop Rare Compound Mutations After Later-Line Osimertinib Treatment
Source: Front Oncol. 2021 Apr 15;11:649843. doi: 10.3389/fonc.2021.649843 (PMC8082017; doi:10.3389/fonc.2021.649843)
Supplement: Supplementary file 1 [file Presentation_1.pptx]

## Slide 1
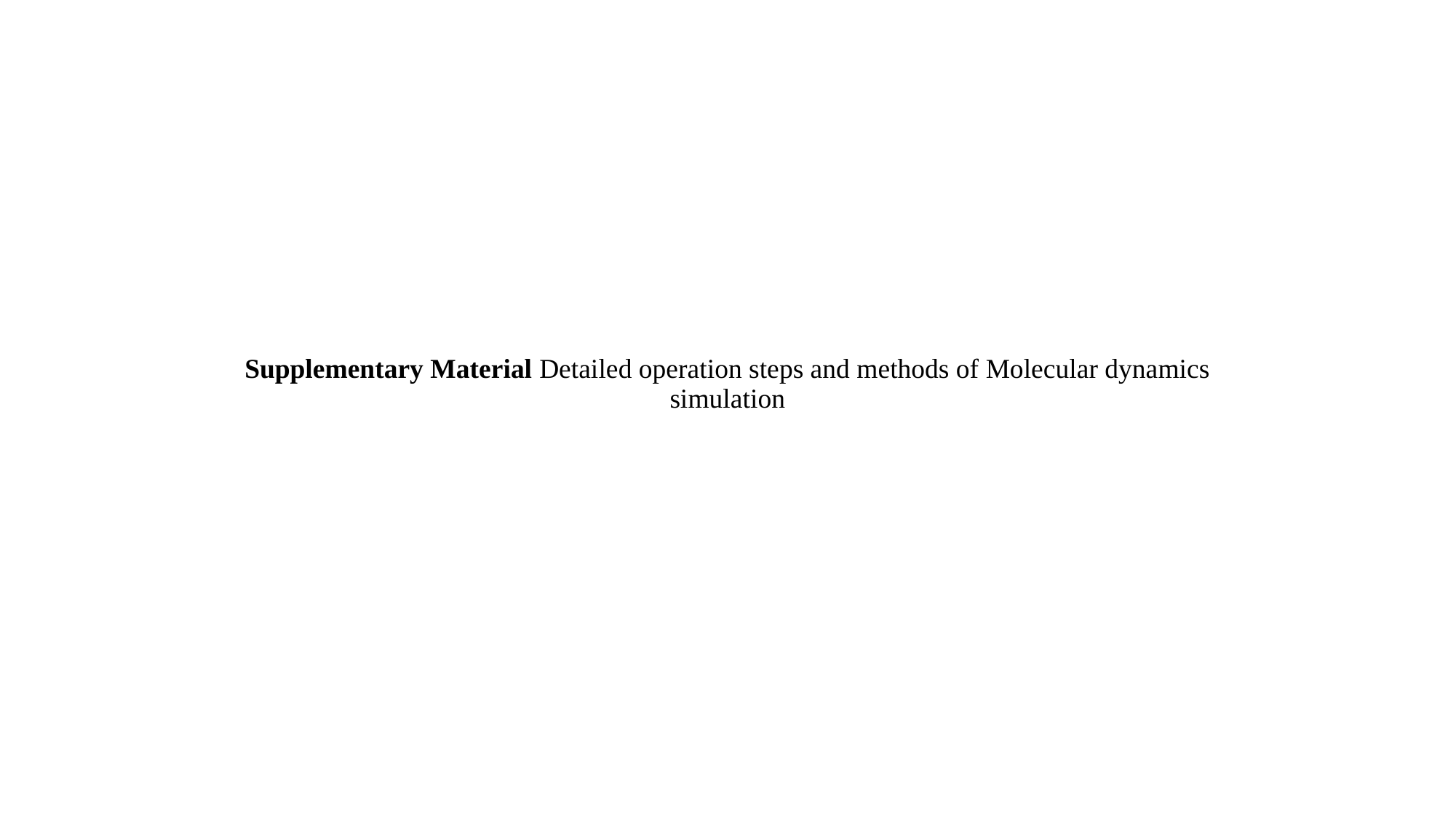

# Supplementary Material Detailed operation steps and methods of Molecular dynamics simulation

## Slide 2
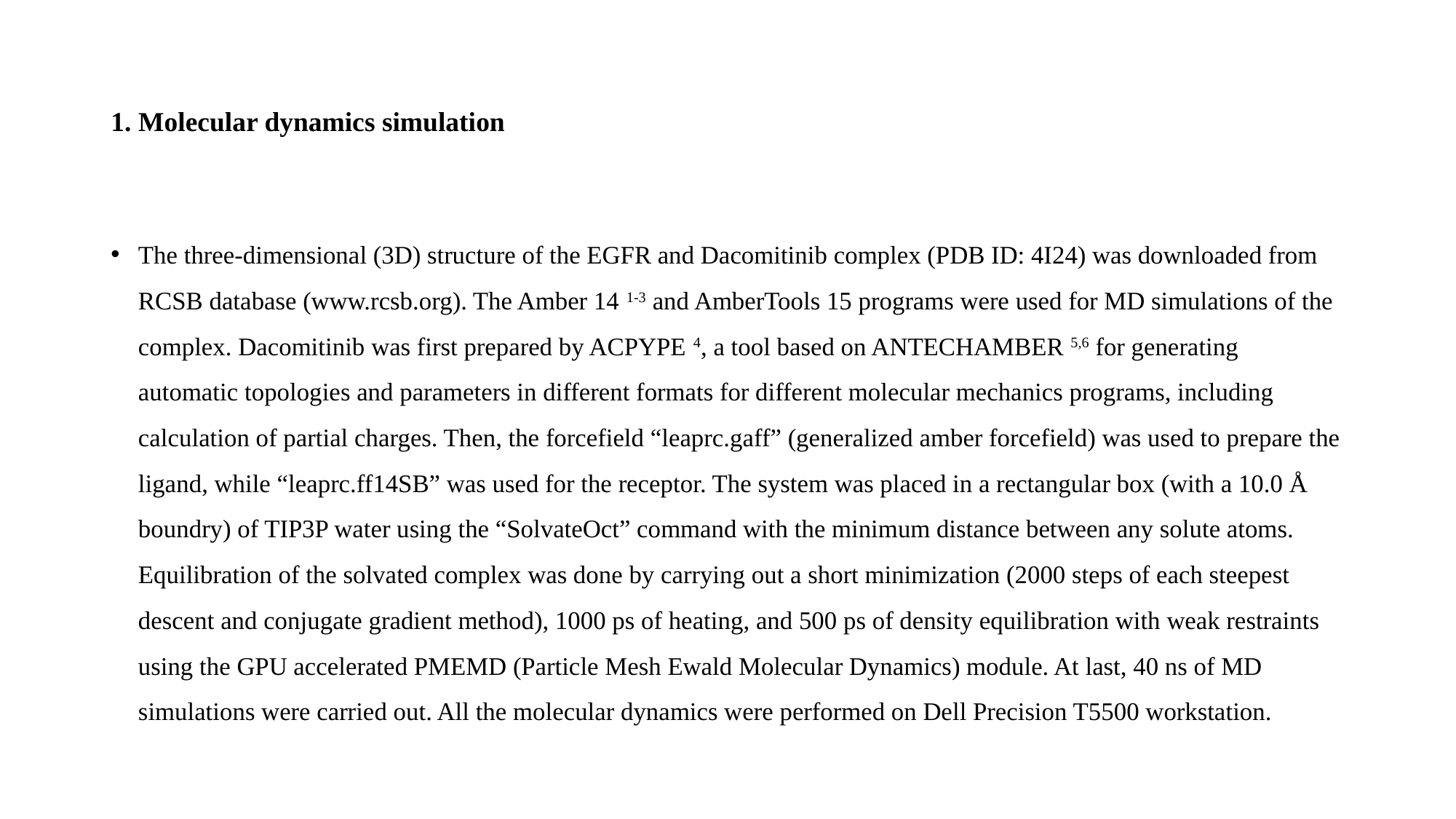

# 1. Molecular dynamics simulation
The three-dimensional (3D) structure of the EGFR and Dacomitinib complex (PDB ID: 4I24) was downloaded from RCSB database (www.rcsb.org). The Amber 14 1-3 and AmberTools 15 programs were used for MD simulations of the complex. Dacomitinib was first prepared by ACPYPE 4, a tool based on ANTECHAMBER 5,6 for generating automatic topologies and parameters in different formats for different molecular mechanics programs, including calculation of partial charges. Then, the forcefield “leaprc.gaff” (generalized amber forcefield) was used to prepare the ligand, while “leaprc.ff14SB” was used for the receptor. The system was placed in a rectangular box (with a 10.0 Å boundry) of TIP3P water using the “SolvateOct” command with the minimum distance between any solute atoms. Equilibration of the solvated complex was done by carrying out a short minimization (2000 steps of each steepest descent and conjugate gradient method), 1000 ps of heating, and 500 ps of density equilibration with weak restraints using the GPU accelerated PMEMD (Particle Mesh Ewald Molecular Dynamics) module. At last, 40 ns of MD simulations were carried out. All the molecular dynamics were performed on Dell Precision T5500 workstation.

## Slide 3
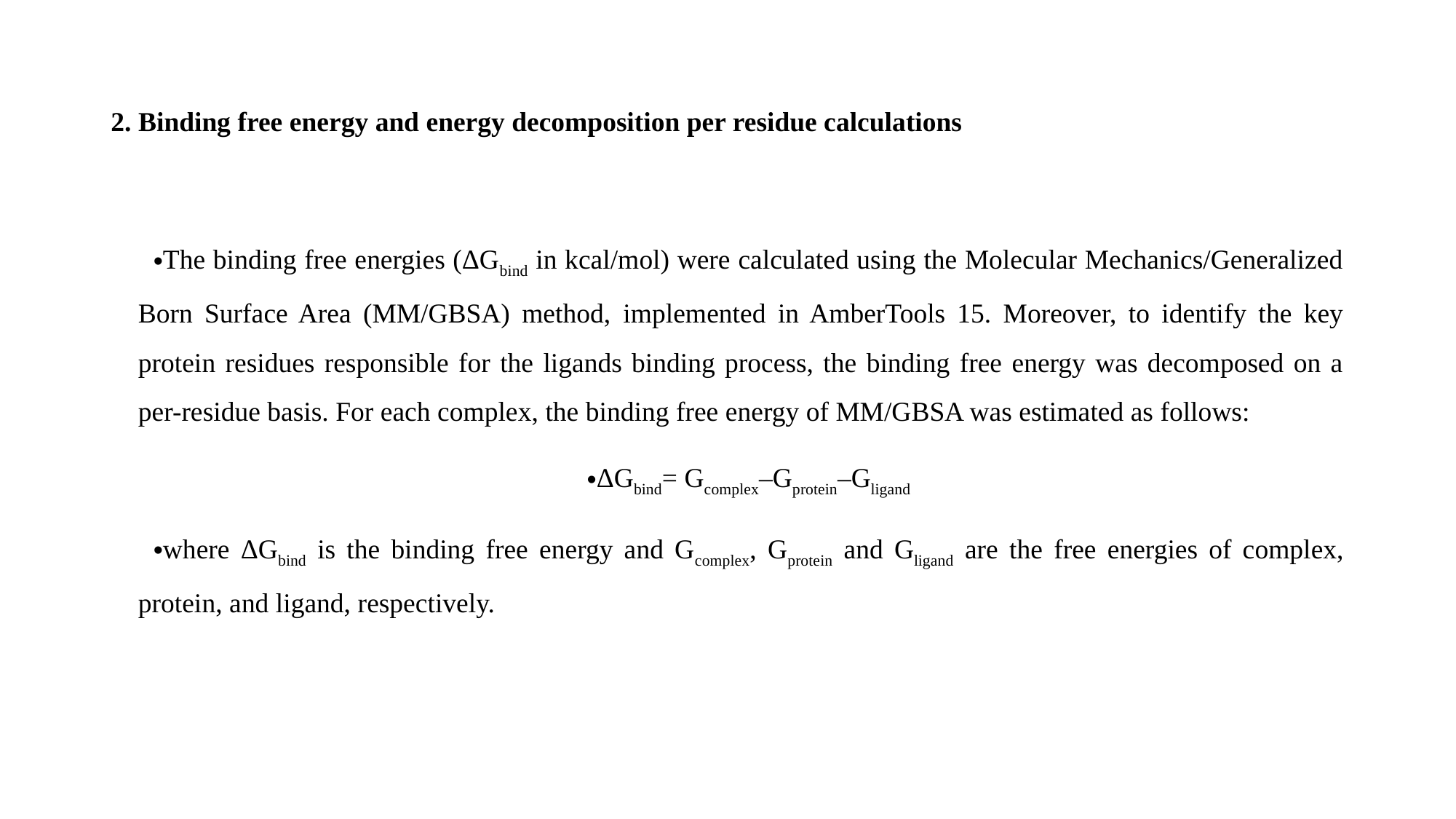

# 2. Binding free energy and energy decomposition per residue calculations
The binding free energies (ΔGbind in kcal/mol) were calculated using the Molecular Mechanics/Generalized Born Surface Area (MM/GBSA) method, implemented in AmberTools 15. Moreover, to identify the key protein residues responsible for the ligands binding process, the binding free energy was decomposed on a per-residue basis. For each complex, the binding free energy of MM/GBSA was estimated as follows:
ΔGbind= Gcomplex‒Gprotein‒Gligand
where ΔGbind is the binding free energy and Gcomplex, Gprotein and Gligand are the free energies of complex, protein, and ligand, respectively.

## Slide 4
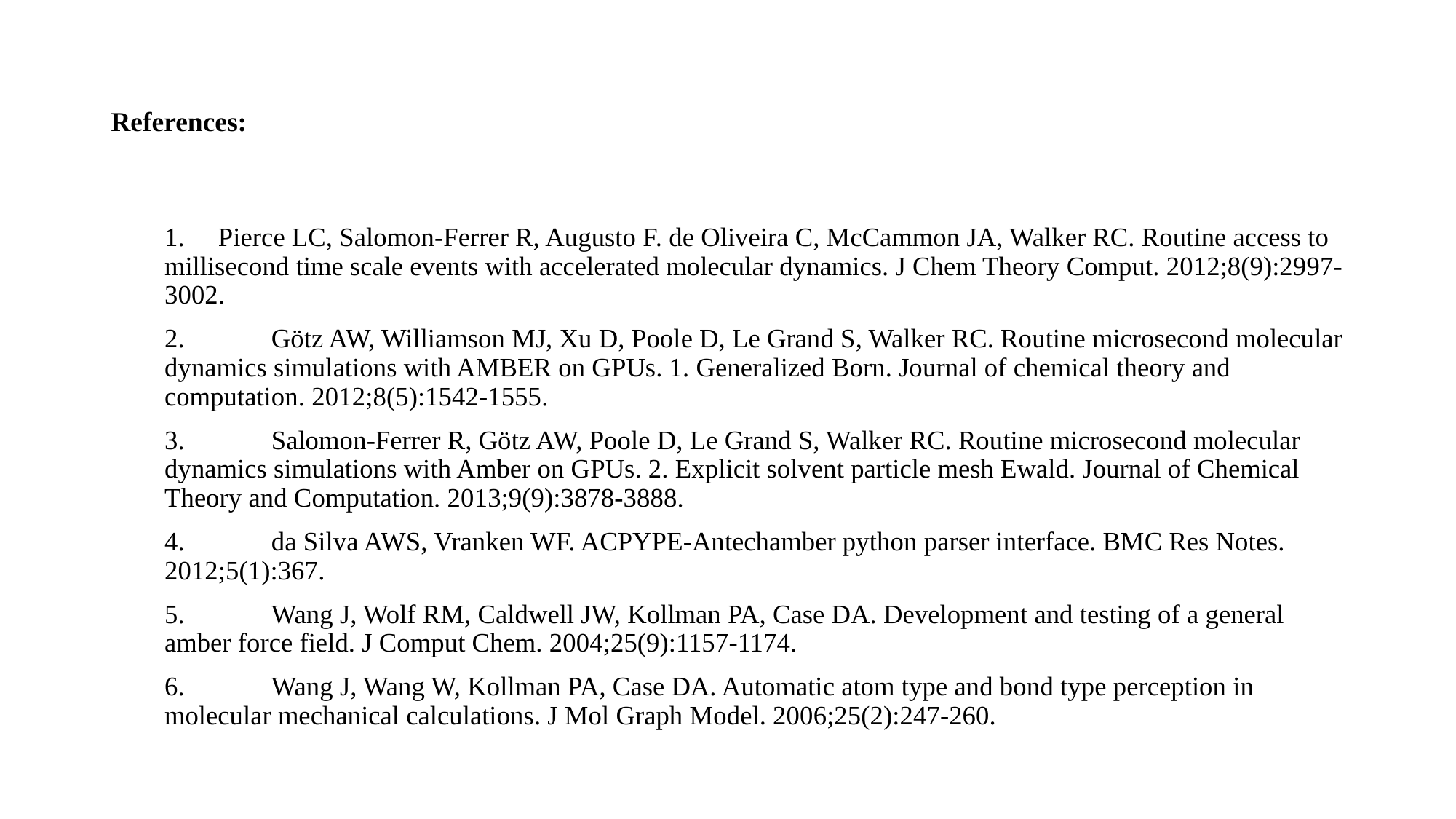

# References:
1. Pierce LC, Salomon-Ferrer R, Augusto F. de Oliveira C, McCammon JA, Walker RC. Routine access to millisecond time scale events with accelerated molecular dynamics. J Chem Theory Comput. 2012;8(9):2997-3002.
2.	Götz AW, Williamson MJ, Xu D, Poole D, Le Grand S, Walker RC. Routine microsecond molecular dynamics simulations with AMBER on GPUs. 1. Generalized Born. Journal of chemical theory and computation. 2012;8(5):1542-1555.
3.	Salomon-Ferrer R, Götz AW, Poole D, Le Grand S, Walker RC. Routine microsecond molecular dynamics simulations with Amber on GPUs. 2. Explicit solvent particle mesh Ewald. Journal of Chemical Theory and Computation. 2013;9(9):3878-3888.
4.	da Silva AWS, Vranken WF. ACPYPE-Antechamber python parser interface. BMC Res Notes. 2012;5(1):367.
5.	Wang J, Wolf RM, Caldwell JW, Kollman PA, Case DA. Development and testing of a general amber force field. J Comput Chem. 2004;25(9):1157-1174.
6.	Wang J, Wang W, Kollman PA, Case DA. Automatic atom type and bond type perception in molecular mechanical calculations. J Mol Graph Model. 2006;25(2):247-260.
