# Supplementary material for: Case Report: Dacomitinib May Not Benefit Patients Who Develop Rare Compound Mutations After Later-Line Osimertinib Treatment
Source: Front Oncol. 2021 Apr 15;11:649843. doi: 10.3389/fonc.2021.649843 (PMC8082017; doi:10.3389/fonc.2021.649843)
Supplement: Supplementary file 2 [file Table_1.docx]

**Supplementary Table 1.** Patients mutation profile besides *EGFR*

| Case | Gender | Age | Co-mutation (abundance) | NGS sample type |
| --- | --- | --- | --- | --- |
| 1 | F | 38 | NA | Blood |
| 2 | M | 45 | *TP53* p.K320Efs*17 (3.05%), *ATRX* p.A419E (0.54%), *BTG1* p.L26P (0.8%), *CLEC9A* p.H2N (0.61%),  *GNAS* p.R202C (0.91%), *LYN* p.T281S (1.45%), *NBN* p.G137A (0.7%),  *RB1* p.C169* (6.92%), *TP53* p.K320Efs*17(3.05%), *TSC2* p.L737F (2.38%) | Blood |
| 3 | M | 58 | NA | Blood |
| 4 | F | 72 | *mTOR* p.P1116R (0.2%)/ *AKT3*^amp^ (1.8), *CDK4*^amp^ (1.9), *GNAS*^amp^ (2.4), *mTOR* p.P1116R (16.7%) | Blood/ pleural effusion |
| 5 | M | 81 | TP53 p.R282W (0.3%) | Liver tissue |

F, female; M, male; NA, not available; NGS, next-generation sequencing; amp, amplification.
