# Supplementary material for: Case Report: Dacomitinib May Not Benefit Patients Who Develop Rare Compound Mutations After Later-Line Osimertinib Treatment
Source: Front Oncol. 2021 Apr 15;11:649843. doi: 10.3389/fonc.2021.649843 (PMC8082017; doi:10.3389/fonc.2021.649843)
Supplement: Supplementary file 3 [file Table_2.docx]

**Supplementary Table 2.** Published NSCLC cases carrying L718 mutation benefited from afatinib

| Authors | Year | Gender | Age | EGFR initial mutation (persistence at the progression to osimertinib) | p.T790M status at the progression to osimertinib | Resistance mechanism to osimertinib (abundance) | NGS sample type | PFS (months) | Best efficacy |
| --- | --- | --- | --- | --- | --- | --- | --- | --- | --- |
| Liu et al. ^1^ | 2019 | Female | 65 | L858R (Y) | Lost | *EGFR* L718Q (44%)、*BRAF* G466R (1%) | Cerebrospinal fluid | 4 | SD |
| Lee et al. ^2^ | 2019 | Female | 53 | L858R (Y) | Lost | *EGFR* L718Q (7%)、*PIK3CA* E545K (12.9%) | Tissue | 3 | PR |
| Fang et al. ^3^ | 2019 | Male | 45 | L858R (Y) | Lost | *EGFR* L718V (82.05%)、*EGFR* amplication | Tissue | 6+ | PR |
| Starrett et al. ^4^ | 2020 | Female | 62 | L858R (Y) | Lost | *EGFR* L718Q (73%)、*EGFR* L718V (3%) | Tissue | 4.5 | PR |
| Yang et al. ^5^ | 2020 | Female | 69 | L858R (Y) | Lost | *EGFR* L718Q (10.8%) | Pleural effusion | 4 | PR |

NSCLC, non-small cell lung cancer; Y, yes; PFS, progression-free survival; PR, partial response; SD, stable disease.

1. Liu J, Jin B, Su H, Qu X, Liu Y. Afatinib helped overcome subsequent resistance to osimertinib in a patient with NSCLC having leptomeningeal metastasis baring acquired EGFR L718Q mutation: a case report. *BMC cancer.* 2019;19(1):702.

2. Lee J, Shim JH, Park WY, et al. Rare mechanism of acquired resistance to osimertinib in Korean patients with EGFR-mutated non-small cell lung cancer. *Cancer Research and Treatment.* 2019;51(1):408-412.

3. Fang W, Gan J, Huang Y, Zhou H, Zhang L. Acquired EGFR L718V Mutation and Loss of T790M-Mediated Resistance to Osimertinib in a Patient With NSCLC Who Responded to Afatinib. *Journal of Thoracic Oncology.* 2019;14(12):e274-e275.

4. Starrett JH, Guernet AA, Cuomo ME, et al. Drug Sensitivity and Allele Specificity of First-Line Osimertinib Resistance Mutations. *Cancer research.* 2020;80(10):2017-2030.

5. Yang X, Huang C, Chen R, Zhao J. Resolving Resistance to Osimertinib Therapy With Afatinib in an NSCLC Patient With EGFR L718Q Mutation. *Clin Lung Cancer.* 2020;21(4):e258-e260.
